# 4-Benz­yloxy-2-bromo-1-meth­oxy­benzene

**DOI:** 10.1107/S1600536811031989

**Published:** 2011-08-11

**Authors:** Xing Huang, Li-Hong Ren, Rong-Hua Yin, Feng Gao

**Affiliations:** a Department of Chinese Traditional Herbal, Agronomy College, Sichuan Agriculture University, Chengdu 611130, People’s Republic of China

## Abstract

In the title compound, C_14_H_13_BrO_2_, the phenyl ring is oriented at a dihedral angle of 72.6 (3)° with respect to the bromo­meth­oxy­phenyl ring. The crystal structure is stabilized by weak inter­molecular C—H⋯O inter­actions.

## Related literature

For the synthesis of analogues of the title compound, see: Shi *et al.* (2004 ▶). The title compound could be converted to aromatic boric acid derivatives, which are significant inter­mediates of various novel bioactive compounds through Suzuki–Miyaura Coupling, see: Suzuki (2011 ▶).
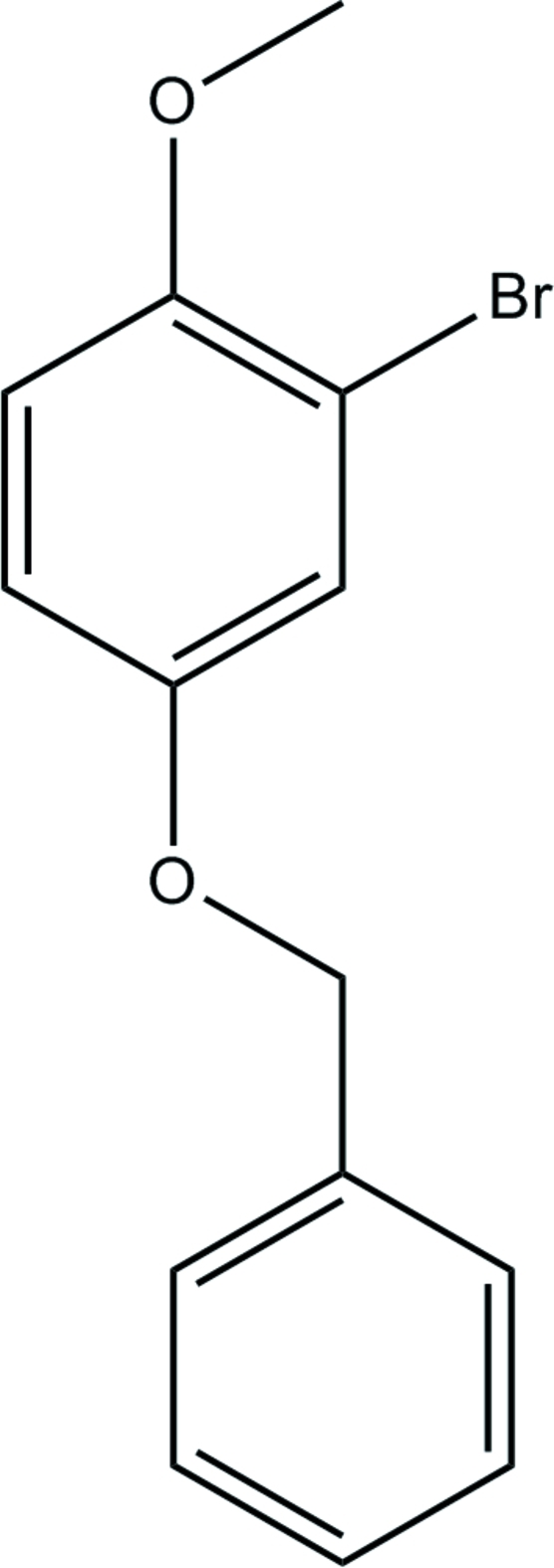


## Experimental

### 

#### Crystal data

C_14_H_13_BrO_2_
*M* *_r_* = 293.15
Monoclinic, 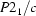

*a* = 6.1415 (7) Å
*b* = 8.2635 (7) Å
*c* = 25.287 (2) Å
β = 94.401 (10)°
*V* = 1279.5 (2) Å^3^
*Z* = 4
Mo *K*α radiation
μ = 3.20 mm^−1^
*T* = 293 K
0.32 × 0.28 × 0.22 mm

#### Data collection

Oxford Diffraction Xcalibur Eos diffractometer
Absorption correction: multi-scan (*CrysAlis PRO*; Oxford Diffraction, 2010 ▶) *T*_min_ = 0.859, *T*_max_ = 1.0
3982 measured reflections
3982 independent reflections
2610 reflections with *I* > 2σ(*I*)

#### Refinement

*R*[*F* ^2^ > 2σ(*F* ^2^)] = 0.055
*wR*(*F* ^2^) = 0.164
*S* = 1.00
3982 reflections
156 parameters
H-atom parameters constrained
Δρ_max_ = 0.45 e Å^−3^
Δρ_min_ = −0.42 e Å^−3^

### 

Data collection: *CrysAlis PRO CCD* (Oxford Diffraction, 2010 ▶); cell refinement: *CrysAlis PRO CCD*; data reduction: *CrysAlis PRO CCD*; program(s) used to solve structure: *SHELXS97* (Sheldrick, 2008 ▶); program(s) used to refine structure: *SHELXL97* (Sheldrick, 2008 ▶); molecular graphics: *OLEX2* (Dolomanov *et al.*, 2009 ▶); software used to prepare material for publication: *publCIF* (Westrip, 2010 ▶).

## Supplementary Material

Crystal structure: contains datablock(s) I, global. DOI: 10.1107/S1600536811031989/xu5279sup1.cif

Structure factors: contains datablock(s) I. DOI: 10.1107/S1600536811031989/xu5279Isup2.hkl

Supplementary material file. DOI: 10.1107/S1600536811031989/xu5279Isup3.cml

Additional supplementary materials:  crystallographic information; 3D view; checkCIF report

## Figures and Tables

**Table 1:** Hydrogen-bond geometry (Å, °)

*D*—H⋯*A*	*D*—H	H⋯*A*	*D*⋯*A*	*D*—H⋯*A*
C5—H5⋯O2^i^	0.93	2.54	3.453 (7)	169

Symmetry code: (i) 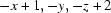.

**Fig. 1.**
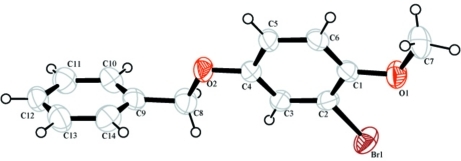
Molecular structre showing 30% probability displacement ellipsoids.

**Fig. 2.**
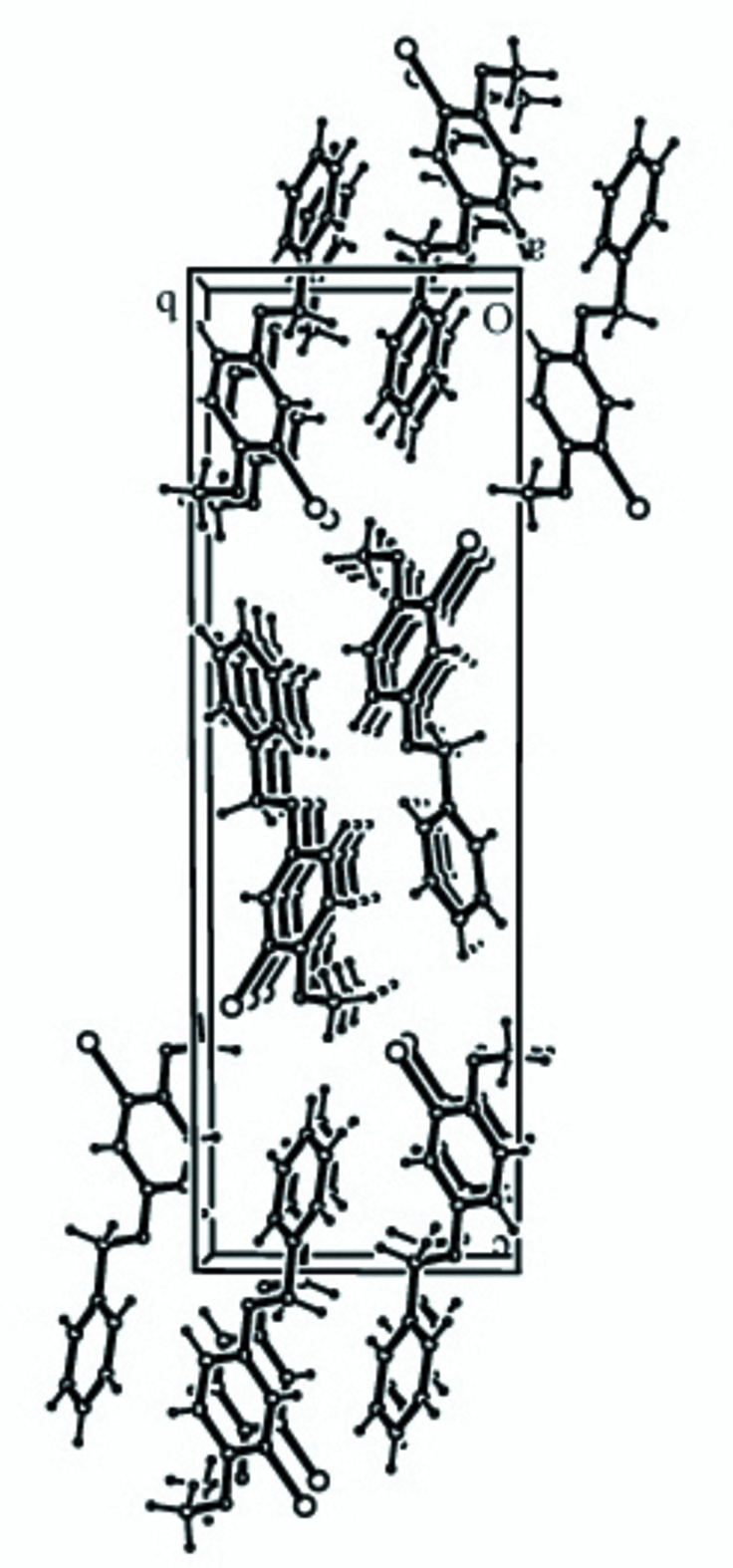
The packing viewed along c axis with C—H···O interactions, indicating the dimer.

## supplementary crystallographic information

### Comment

The title compound, 4-(benzyloxy)-2-bromo-1-methoxybenzene was synthesize from 4-methoxyphenol through 4 steps reactions. The hydroxyl group of 4-methoxyphenol was protected by acetyl to give 4-methoxyphenyl acetate. Then *ortho*-position aromatic hydrogen atom of methoxy was substituted by bromidum to abtain 3-bromo-4-methoxyphenyl acetate when NBS (*N*-bromosuccinimide) was added in CH_3_CN. After hydrolysis of acetyl group and re-protection by benzyl group with benzyl bromide, the title compound was prerarated almost quantitatively.

4-(Benzyloxy)-2-bromo-1-methoxybenzene could be converted to aromatic boric acid derivates, which are significant intermediate to form various novel bioactive compounds throngh Suzuki–Miyaura Coupling. Herein, we report the crystal structure of the important compound.

The title compound have two armatic rings, which are nearly orthogonal to each other [dihedral angle 72.58°]. The central oxygen atom (O_2_) and carbon atom (C_8_) are nearly coplanar with the bromobenzoyl ring and the benzoyl rings [O_2_—C_4_—C_5_—C_6_ torsion angles = 178.5 (6)° and C_8_—C_9_—C_10_—C_11_ torsion angles = 176.5 (6)°], respectively. The crystal structure is stabilized by weak intermolecular C—H···O interactions (Table 1).

### Experimental

Single crystals of 4-(benzyloxy)-2-bromo-1-methoxybenzene, C_14_H_13_BrO_2_ were recrystallized from acetone mounted in inert oil and transferred to the cold gas stream of the diffractometer.

### Refinement

All the H-atoms were placed in calculated positions and treated as riding atoms [C—H = 0.93 - 0.96 Å], with U_iso_(H) = 1.5U_eq_(C) for methyl H atoms and 1.2U_eq_(C) for the others.

### Crystal data

**Table:**

C_14_H_13_BrO_2_	*F*(000) = 592
*M**_r_* = 293.15	*D*_x_ = 1.522 Mg m^−^^3^
Monoclinic, *P*2_1_/*c*	Mo *K*α radiation, λ = 0.7107 Å
Hall symbol: -P 2ybc	Cell parameters from 1416 reflections
*a* = 6.1415 (7) Å	θ = 2.9–26.3°
*b* = 8.2635 (7) Å	µ = 3.20 mm^−^^1^
*c* = 25.287 (2) Å	*T* = 293 K
β = 94.401 (10)°	Block, colourless
*V* = 1279.5 (2) Å^3^	0.32 × 0.28 × 0.22 mm
*Z* = 4	

### Data collection

**Table:**

Oxford Diffraction Xcalibur Eos diffractometer	3982 independent reflections
Radiation source: fine-focus sealed tube	2610 reflections with *I* > 2σ(*I*)
graphite	*R*_int_ = 0.000
Detector resolution: 16.0874 pixels mm^-1^	θ_max_ = 26.4°, θ_min_ = 3.0°
ω scans	*h* = −7→7
Absorption correction: multi-scan (*CrysAlis PRO*; Oxford Diffraction, 2010)	*k* = −10→10
*T*_min_ = 0.859, *T*_max_ = 1.0	*l* = −30→31
3982 measured reflections	

### Refinement

**Table:**

Refinement on *F*^2^	Primary atom site location: structure-invariant direct methods
Least-squares matrix: full	Secondary atom site location: difference Fourier map
*R*[*F*^2^ > 2σ(*F*^2^)] = 0.055	Hydrogen site location: inferred from neighbouring sites
*wR*(*F*^2^) = 0.164	H-atom parameters constrained
*S* = 1.00	*w* = 1/[σ^2^(*F*_o_^2^) + (0.1035*P*)^2^] where *P* = (*F*_o_^2^ + 2*F*_c_^2^)/3
3982 reflections	(Δ/σ)_max_ = 0.001
156 parameters	Δρ_max_ = 0.45 e Å^−^^3^
0 restraints	Δρ_min_ = −0.42 e Å^−^^3^

### Special details

**Table:**

Geometry. All e.s.d.'s (except the e.s.d. in the dihedral angle between two l.s. planes) are estimated using the full covariance matrix. The cell e.s.d.'s are taken into account individually in the estimation of e.s.d.'s in distances, angles and torsion angles; correlations between e.s.d.'s in cell parameters are only used when they are defined by crystal symmetry. An approximate (isotropic) treatment of cell e.s.d.'s is used for estimating e.s.d.'s involving l.s. planes.
Refinement. Refinement of *F*^2^ against ALL reflections. The weighted *R*-factor *wR* and goodness of fit *S* are based on *F*^2^, conventional *R*-factors *R* are based on *F*, with *F* set to zero for negative *F*^2^. The threshold expression of *F*^2^ > σ(*F*^2^) is used only for calculating *R*-factors(gt) *etc*. and is not relevant to the choice of reflections for refinement. *R*-factors based on *F*^2^ are statistically about twice as large as those based on *F*, and *R*- factors based on ALL data will be even larger.

### Fractional atomic coordinates and isotropic or equivalent isotropic displacement parameters (Å^2^)

**Table:**

	*x*	*y*	*z*	*U*_iso_*/*U*_eq_	
Br1	0.52954 (11)	0.35775 (8)	0.77207 (3)	0.0720 (3)	
O1	0.1613 (7)	0.1331 (5)	0.78343 (15)	0.0634 (11)	
O2	0.6766 (7)	0.1821 (5)	0.97133 (16)	0.0691 (13)	
C1	0.2856 (9)	0.1387 (7)	0.8308 (2)	0.0509 (14)	
C2	0.4647 (9)	0.2388 (6)	0.8329 (2)	0.0478 (14)	
C3	0.6002 (10)	0.2562 (6)	0.8784 (2)	0.0535 (16)	
H3	0.7197	0.3254	0.8788	0.064*	
C4	0.5592 (11)	0.1714 (6)	0.9233 (2)	0.0547 (16)	
C5	0.3786 (10)	0.0679 (7)	0.9211 (2)	0.0605 (16)	
H5	0.3478	0.0097	0.9511	0.073*	
C6	0.2472 (10)	0.0511 (6)	0.8754 (2)	0.0548 (16)	
H6	0.1301	−0.0204	0.8744	0.066*	
C7	−0.0252 (9)	0.0291 (9)	0.7806 (2)	0.080 (2)	
H7A	−0.1048	0.0410	0.7467	0.120*	
H7C	−0.1177	0.0575	0.8081	0.120*	
H7B	0.0217	−0.0812	0.7852	0.120*	
C8	0.8485 (11)	0.2936 (8)	0.9758 (3)	0.0695 (19)	
H8B	0.7976	0.3985	0.9628	0.083*	
H8A	0.9648	0.2583	0.9546	0.083*	
C9	0.9321 (11)	0.3061 (7)	1.0329 (2)	0.0573 (16)	
C10	1.1363 (11)	0.2430 (7)	1.0508 (3)	0.0678 (19)	
H10	1.2196	0.1869	1.0276	0.081*	
C11	1.2111 (11)	0.2643 (8)	1.1021 (3)	0.072 (2)	
H11	1.3478	0.2234	1.1135	0.087*	
C12	1.0936 (13)	0.3434 (8)	1.1375 (3)	0.0714 (19)	
H12	1.1467	0.3536	1.1728	0.086*	
C13	0.8960 (13)	0.4075 (7)	1.1202 (3)	0.075 (2)	
H13	0.8154	0.4652	1.1435	0.091*	
C14	0.8152 (12)	0.3870 (7)	1.0678 (3)	0.0700 (19)	
H14	0.6792	0.4292	1.0566	0.084*	

### Atomic displacement parameters (Å^2^)

**Table:**

	*U*^11^	*U*^22^	*U*^33^	*U*^12^	*U*^13^	*U*^23^
Br1	0.0649 (4)	0.0851 (4)	0.0653 (4)	−0.0096 (4)	0.0005 (4)	0.0306 (4)
O1	0.058 (3)	0.078 (3)	0.051 (2)	−0.019 (2)	−0.015 (2)	0.005 (2)
O2	0.085 (3)	0.067 (3)	0.052 (3)	−0.034 (2)	−0.016 (2)	0.009 (2)
C1	0.052 (4)	0.045 (3)	0.056 (4)	−0.002 (3)	0.003 (3)	−0.008 (3)
C2	0.050 (4)	0.043 (3)	0.050 (4)	0.001 (3)	0.003 (3)	0.003 (3)
C3	0.056 (4)	0.045 (3)	0.059 (4)	−0.007 (3)	0.002 (3)	0.006 (3)
C4	0.070 (4)	0.047 (3)	0.047 (4)	−0.007 (3)	0.001 (3)	0.000 (3)
C5	0.081 (5)	0.052 (3)	0.049 (4)	−0.021 (4)	0.009 (4)	0.000 (3)
C6	0.060 (4)	0.050 (3)	0.054 (4)	−0.018 (3)	0.001 (3)	−0.003 (3)
C7	0.071 (5)	0.102 (5)	0.067 (5)	−0.024 (4)	0.004 (4)	−0.010 (4)
C8	0.071 (5)	0.073 (4)	0.065 (5)	−0.023 (4)	0.005 (4)	0.003 (4)
C9	0.064 (4)	0.053 (3)	0.055 (4)	−0.021 (3)	0.006 (4)	−0.002 (3)
C10	0.059 (4)	0.070 (4)	0.075 (5)	0.000 (4)	0.008 (4)	−0.017 (4)
C11	0.056 (4)	0.079 (5)	0.079 (5)	0.003 (4)	−0.014 (4)	−0.007 (4)
C12	0.093 (6)	0.065 (4)	0.054 (4)	−0.018 (5)	−0.006 (4)	−0.004 (4)
C13	0.083 (6)	0.070 (4)	0.075 (5)	−0.008 (4)	0.015 (4)	−0.018 (4)
C14	0.059 (4)	0.074 (4)	0.076 (5)	0.009 (4)	−0.003 (4)	0.003 (4)

### Geometric parameters (Å, °)

**Table:**

Br1—C2	1.893 (5)	C7—H7B	0.9600
O1—C1	1.370 (6)	C8—H8B	0.9700
O1—C7	1.430 (7)	C8—H8A	0.9700
O2—C4	1.367 (7)	C8—C9	1.499 (8)
O2—C8	1.399 (7)	C9—C10	1.401 (9)
C1—C2	1.374 (7)	C9—C14	1.356 (9)
C1—C6	1.377 (8)	C10—H10	0.9300
C2—C3	1.375 (8)	C10—C11	1.354 (9)
C3—H3	0.9300	C11—H11	0.9300
C3—C4	1.374 (8)	C11—C12	1.360 (9)
C4—C5	1.398 (8)	C12—H12	0.9300
C5—H5	0.9300	C12—C13	1.365 (9)
C5—C6	1.364 (8)	C13—H13	0.9300
C6—H6	0.9300	C13—C14	1.387 (9)
C7—H7A	0.9600	C14—H14	0.9300
C7—H7C	0.9600		
			
O1—C1—C2	116.4 (5)	C6—C5—H5	119.6
O1—C1—C6	125.4 (5)	H7A—C7—H7C	109.5
O1—C7—H7A	109.5	H7A—C7—H7B	109.5
O1—C7—H7C	109.5	H7C—C7—H7B	109.5
O1—C7—H7B	109.5	H8B—C8—H8A	108.3
O2—C4—C3	125.7 (6)	C9—C8—H8B	109.9
O2—C4—C5	116.0 (5)	C9—C8—H8A	109.9
O2—C8—H8B	109.9	C9—C10—H10	120.3
O2—C8—H8A	109.9	C9—C14—C13	120.9 (7)
O2—C8—C9	108.9 (5)	C9—C14—H14	119.6
C1—O1—C7	117.0 (4)	C10—C9—C8	121.1 (6)
C1—C2—Br1	119.9 (4)	C10—C11—H11	118.8
C1—C2—C3	121.8 (5)	C10—C11—C12	122.4 (6)
C1—C6—H6	119.6	C11—C10—C9	119.3 (6)
C2—C1—C6	118.1 (5)	C11—C10—H10	120.3
C2—C3—H3	120.0	C11—C12—H12	120.7
C3—C2—Br1	118.3 (4)	C11—C12—C13	118.6 (7)
C3—C4—C5	118.3 (6)	C12—C11—H11	118.8
C4—O2—C8	117.2 (5)	C12—C13—H13	119.9
C4—C3—C2	120.1 (6)	C12—C13—C14	120.2 (7)
C4—C3—H3	120.0	C13—C12—H12	120.7
C4—C5—H5	119.6	C13—C14—H14	119.6
C5—C6—C1	120.9 (5)	C14—C9—C8	120.2 (6)
C5—C6—H6	119.6	C14—C9—C10	118.6 (6)
C6—C5—C4	120.8 (5)	C14—C13—H13	119.9
			
Br1—C2—C3—C4	−179.7 (4)	C6—C1—C2—Br1	178.4 (4)
O1—C1—C2—Br1	−0.7 (7)	C6—C1—C2—C3	−1.8 (8)
O1—C1—C2—C3	179.1 (5)	C7—O1—C1—C2	179.8 (5)
O1—C1—C6—C5	−178.6 (5)	C7—O1—C1—C6	0.9 (8)
O2—C4—C5—C6	178.5 (6)	C8—O2—C4—C3	2.6 (9)
O2—C8—C9—C10	109.0 (7)	C8—O2—C4—C5	−175.4 (5)
O2—C8—C9—C14	−74.4 (7)	C8—C9—C10—C11	176.5 (6)
C1—C2—C3—C4	0.5 (9)	C8—C9—C14—C13	−176.3 (6)
C2—C1—C6—C5	2.4 (9)	C9—C10—C11—C12	1.0 (11)
C2—C3—C4—O2	−177.7 (5)	C10—C9—C14—C13	0.2 (9)
C2—C3—C4—C5	0.3 (9)	C10—C11—C12—C13	−2.0 (11)
C3—C4—C5—C6	0.3 (9)	C11—C12—C13—C14	2.1 (10)
C4—O2—C8—C9	170.9 (5)	C12—C13—C14—C9	−1.3 (10)
C4—C5—C6—C1	−1.7 (9)	C14—C9—C10—C11	−0.1 (9)

### Hydrogen-bond geometry (Å, °)

**Table:**

*D*—H···*A*	*D*—H	H···*A*	*D*···*A*	*D*—H···*A*
C5—H5···O2^i^	0.93	2.54	3.453 (7)	169

Symmetry codes: (i) −*x*+1, −*y*, −*z*+2.
